# An Improved Slice Reconciliation Protocol for Continuous-Variable Quantum Key Distribution

**DOI:** 10.3390/e23101317

**Published:** 2021-10-09

**Authors:** Xuan Wen, Qiong Li, Haokun Mao, Xiaojun Wen, Nan Chen

**Affiliations:** 1 Department of Computer Science and Technology, Harbin Institute of Technology, Harbin 150000, China; wenxuan@hit.edu.cn (X.W.); 14B303003@hit.edu.cn (H.M.); 2 School of Electronics and Information Engineering, Shenzhen Polytechnic, Shenzhen 518000, China; 3 School of Foreign Languages, Harbin Institute of Technology, Harbin 150000, China; chennan2010@hit.edu.cn

**Keywords:** continuous-variable quantum key distribution, reconciliation, slice error correction, polar codes, finite-size effect

## Abstract

Reconciliation is an essential procedure for continuous-variable quantum key distribution (CV-QKD). As the most commonly used reconciliation protocol in short-distance CV-QKD, the slice error correction (SEC) allows a system to distill more than 1 bit from each pulse. However, the quantization efficiency is greatly affected by the noisy channel with a low signal-to-noise ratio (SNR), which usually limits the secure distance to about 30 km. In this paper, an improved SEC protocol, named Rotated-SEC (RSEC), is proposed through performing a random orthogonal rotation on the raw data before quantization, and deducing a new estimator for the quantized sequences. Moreover, the RSEC protocol is implemented with polar codes. The experimental results show that the proposed protocol can reach up to a quantization efficiency of about 99%, and maintain at around 96% even at the relatively low SNRs (0.5,1), which theoretically extends the secure distance to about 45 km. When implemented with the polar codes with a block length of 16 Mb, the RSEC achieved a reconciliation efficiency of above 95%, which outperforms all previous SEC schemes. In terms of finite-size effects, we achieved a secret key rate of $7.83\times 10^{-3}$ bits/pulse at a distance of 33.93 km (the corresponding SNR value is 1). These results indicate that the proposed protocol significantly improves the performance of SEC and is a competitive reconciliation scheme for the CV-QKD system.

## 1. Introduction

Quantum key distribution (QKD), which enables two remote legitimate parties (i.e., Alice and Bob) to share unconditional secret keys against a potential eavesdropper, is a major practical quantum cryptography technology in quantum information [1]. There are mainly two categories of QKD protocols, namely discrete-variable (DV) protocols [2,3,4,5,6] and continuous-variable (CV) protocols [7,8,9,10,11], which, respectively, encode information on discrete variables (such as the polarization or the phase of single photons) and continuous variables (such as the quadratures of coherent states).

The DV-QKD needs a high-cost single-photon detector requiring cryogenic temperatures to measure the received quantum state, which presents a challenge for its widespread implementation. Compared to the DV-QKD, the CV-QKD takes the advantage of using a standard and cost-effective detector that is routinely deployed in standard telecom components working at room temperature. The security proof of CV-QKD against general attacks has been provided [12,13,14,15]. Many experiments of CV-QKD have been successfully implemented, especially the integrated silicon photonic chip for CV-QKD that offers new possibilities for low-cost and portable quantum communication [16].

**Figure 1:**
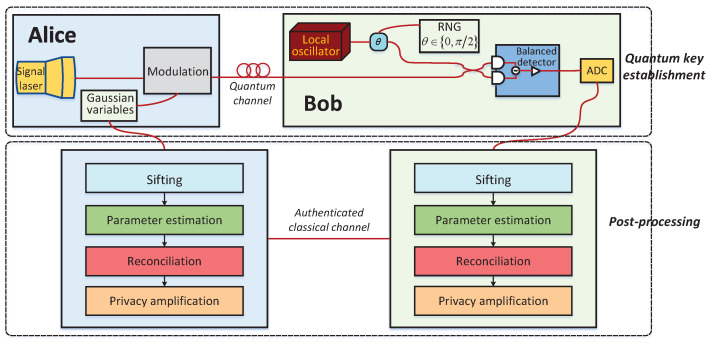
Schematic diagram of the CV-QKD system.

A CV-QKD system mainly includes two consecutive phases [7,8,9]: the quantum key establishment phase and the classical post-processing phase, which are illustrated in Figure 1. In the first phase, Alice prepares a coherent state using two Gaussian variables and sends it to Bob through the quantum channel. Then, Bob randomly chooses one of the two variables to measure his received coherent state and informs Alice of his choice. Owing to the physical noises or the existence of Eve [17] in a quantum channel, the raw data of the legitimate parties obtained from the first phase are weakly correlated and weakly secure continuous variables.

To extract identical secret keys from their raw data, Alice and Bob subsequently perform a phase called post-processing, including four main stages: sifting, parameter estimation [18,19,20], reconciliation [21,22,23,24,25], and privacy amplification [26,27,28]. Reconciliation is a crucial stage for CV-QKD, which allows the legitimate parties to distill the corrected keys from their raw data via an authentic classical channel. Its performance affects the secret key rate and the secure distance of the practical CV-QKD system [29,30,31,32].

Up to now, various reconciliation schemes have been proposed for reconciling the raw data of CV-QKD. Originally, C. Silberhorn et al. proposed sign reconciliation that first quantifies the raw data to bit string by using the sign and then corrects the error bits [21]; however, its low reconciliation efficiency limits its application. Subsequently, V. Assche et al. proposed SEC, which chooses a set of quantization functions to convert a continuous variable into binary-value slices and then executes error correction on the quantized slices [22,23].

Soon after, many researchers apply code-modulated techniques, including multilevel coding (MLC) and multistage decoding (MSD) in SEC with Low Density Parity Check (LDPC) codes to improve the reconciliation performance at high SNRs [33,34]. The SEC scheme allows one to extract more than one bit of key from per pulse, especially at the high SNRs; however, its quantization performance is poor at the low SNR of long-distance CV-QKD, which limits its secure distance to about 30 km. Afterward multidimensional reconciliation was proposed by Anthony Leverrier et al. [24], which extends the secure distance from 30 km to above 50 km. Since the code rate of multidimensional reconciliation is limited to 1 bit per pulse, its related research is mainly focused on improving the reconciliation efficiency with LDPC codes, and especially with Multi-edge type LDPC (MET-LDPC) codes at low SNRs [35,36,37,38,39,40,41].

In summary, the existing research on reconciliation is mainly based on SEC and multidimensional reconciliation. These two schemes have their own advantages and disadvantages. Multidimensional reconciliation has a better quantization scenario than SEC reconciliation, and thus it can still achieve a high-efficiency reconciliation for a long-distance CV-QKD system with a noisy channel. However, its code rate is limited to 1 bit per pulse, which makes it more suitable for a long-distance CV-QKD system. Compared with multidimensional reconciliation, the SEC has advantages in extracting more than 1 bit of secret key per channel use.

Limited by its quantization performance, the SEC protocol is more suitable for the short-distance CV-QKD system. As is known, the secret key rate of a QKD system will decrease rapidly with the increase of distance [29]. Due to the technology immaturity of the physical device, the key generation rate of the long-distance CV-QKD system is generally low [32,42], which obviously cannot satisfy the communication demand.

Therefore, to establish QKD networks [43,44,45,46] with the short-distance QKD system is a practical scheme to provide relatively high-speed keys for secure communication at present [47]. In addition, the LDPC code is usually chosen to pursue a high reconciliation efficiency, but its matrix design is extremely difficult. By contrast, another common family of codes, polar codes, is relatively easier to construct and their recursive structure delivers excellent performance in practice.

In this research, our work focus on the improvement of the SEC protocol and the reconciliation of the data with polar codes. The main contributions of this paper are as follows: (i) We improve the SEC protocol by first performing a random orthogonal rotation on the raw data before slice quantization and then provide a novel estimator for the quantized slices. Compared with the SEC protocol, the improved protocol, named RSEC, has a higher quantization efficiency, which then increases the secret key rate and reconciliation efficiency. (ii) In order to accomplish the reconciliation of the correlated continuous variable in CV-QKD, we implement the RSEC protocol by combining the polar codes, achieving a high-efficiency reconciliation.

The rest of this paper is organized as follows: In Section 2, the RSEC protocol is proposed to improve the SEC protocol. In Section 3, the implementation of the RSEC protocol with polar codes is described. In Section 4, the experimental results and analysis of RSEC are given. Finally, our conclusions are drawn in Section 5.

## 2. Rotated Slice Error Correction (RSEC) Protocol

In this section, we briefly review the SEC reconciliation and then put forward RSEC to improve the current SEC. After the quantum key establishment phase of the Gaussian-modulated coherent state CV-QKD protocol, Alice and Bob share weakly correlated continuous-variable raw data due to the noises during the quantum transmission. The noises can safely be assumed to be Gaussian since they correspond to the case of the optimal attack for Eve [12].

The correlated raw data are obtained by randomly measuring either the the amplitude and phase quadratures for each coherent state. Moreover, the information encoded on the two quadratures follow the same Gaussian distribution. For the convenience of description, let $X=(x_{1},x_{2},\cdots )$ and $Y=(y_{1},y_{2},\cdots )$ correspond to the correlated Gaussian random variables of Alice and Bob, respectively. Then, the correlated raw data can be modeled as Y=X+Z with $x_{i}∼N\left(0,\delta^{2}\right)$, $z_{i}∼N\left(0,\sigma^{2}\right)$, where $Z=(z_{1},z_{2},\cdots )$, $\delta^{2}$ and $\sigma^{2}$ denote Alice’s modulation variance and the noise variance, respectively.

In the direct reconciliation scenario, Alice’s sequence is used as the target to correct Bob’s sequence. On the contrary, the reverse reconciliation scenario uses Bob’s sequence as the target to correct Alice’s sequence. Generally, the latter scenario can obtain a higher secret key rate [35,38]. Without loss of generality, we only consider the reverse reconciliation in this research.

### 2.1. Review of Slice Error Correction

In information reconciliation, Alice and Bob first perform an operation called quantization to convert the correlated values into binary sequences and then choose an error correction scheme to correct the binary sequence over an authenticated classical channel. SEC is a generic reconciliation protocol [22]. Its underlying idea is to convert Alice’s and Bob’s values into bit strings with the slice function (i.e., quantization function) and then apply an error correction scheme as a primitive, taking advantage of all available information to minimize the number of exchanged reconciliation messages.

This works in two steps: First, Bob chooses a quantization function $S\left(x\right):R\to {\left\{0,1\right\}}^{m}$ to map his raw data to *m*-slices binary digits, and informs Alice of the first *t* slices (usually *t* = 2 or 3), S(x) is a vector of slices $S\left(x\right)=(S_{1}\left(x\right),\cdots ,S_{m}\left(x\right))$; then, Bob sequentially deals with the remaining slice *k* (t+1≤k≤m) by sending a syndrome of $S_{k}\left(x\right)$ to Alice so that Alice can recover $S_{k}\left(x\right)$ with a high probability.

The quantization function is to divide the set of real numbers R into $2^{m}$ intervals and then to assign different binary values to each of these intervals. There are two different schemes to construct the quantization function. The first construction scheme is to divide R with $2^{m}-1$ equidistant points. The second construction scheme freely chooses $2^{m}-1$ points to divide R, which performs better but has a much higher computational complexity. The previous work indicated that the second scheme does not improve as much as the quantization efficiency compared with the first scheme [33]. Therefore, we use the first scheme to construct the quantization function in this research.

In addition, previous studies have shown that the best bit assignment method is to assign the least significant bit of the binary representation of a−1 $\left(0\leq a-1\leq 2^{m}-1\right)$ to the first slice $S_{1}\left(x\right)$ when $\tau_{a-1}\leq x<\tau_{a}$ [22]. The variables $\tau_{j}$ divide the real numbers R into $2^{m}$ intervals, where $1\leq j\leq 2^{m}-1$, $\tau_{0}=-\infty$, $\tau_{2^{m}}=+\infty$. Then, each bit of a−1 is subsequently assigned to the remaining slices. More specifically,

(1) $$S_{i}\left(x\right)=\;\left\{\begin{array}{c} 0\;,\mathrm{if}\;\tau_{2^{i}n}\leq x<\tau_{2^{i}n+2^{i-1}}\\ 1\;,\mathrm{otherwise} \end{array}\right.,$$

where 1≤i≤m and *n* is a nonnegative integer.

### 2.2. Improvement of Slice Error Correction

In the decoding process of SEC, the slice sequences are corrected in sequence; hence, the estimation of the current slice recursively depends on all previous slices. For this reason, the performance of SEC can be improved by reducing the bit error rate (BER) $e_{i}$ of the previously decoded slices, $e_{i}$ denotes the probability that Alice makes a wrong estimate of Bob’s slice value $S_{i}\left(x\right)$. According to the characteristics of quantization function S(x), it is not hard to find that the last slice $S_{m}\left(x\right)$ corresponds exactly to the sign of input variable *x*. Therefore, the quantization scheme of the last slice is similar to the multidimensional reconciliation, which uses the sign of the rotated data as the target sequence.

As is known, multidimensional reconciliation typically performs better than the SEC reconciliation in estimating the quantized values, especially at a low SNR [24]. For each slice, although having obtained the first few slices, Alice still needs to infer Bob’s slice value in a certain number of intervals. Taking the case of m=4 slices as an example, if Alice has the first two slices $(S_{1}\left(x\right),S_{2}\left(x\right))=\left(0,1\right)$, she needs to estimate Bob’s slice $S_{3}\left(x\right)$ among four intervals, i.e., $\left(\tau_{2},\tau_{3}\right)$, $\left(\tau_{6},\tau_{7}\right)$, $\left(\tau_{10},\tau_{11}\right)$, $\left(\tau_{14},\tau_{15}\right)$ to satisfy $(S_{1}\left(x\right),S_{2}\left(x\right))=\left(0,1\right)$. However, multidimensional reconciliation calculates the probabilities of Bob’s quantized value with the joint density function directly, which leads to more accurate estimations.

**Figure 2:**
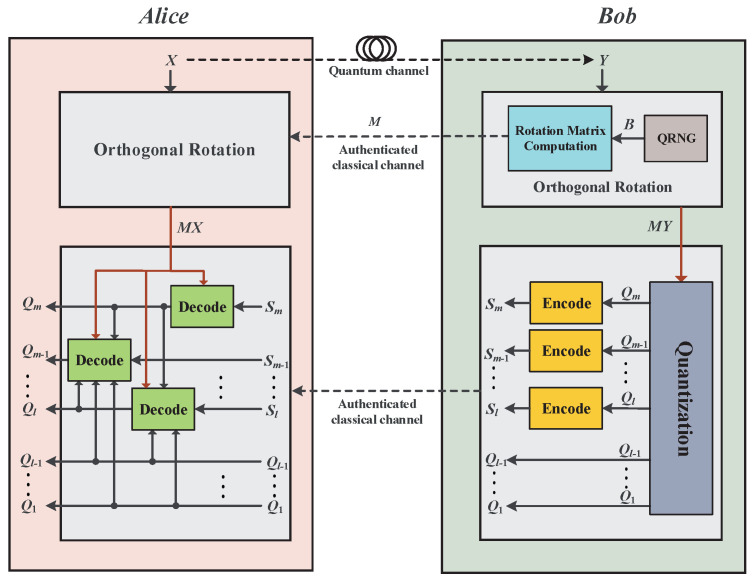
Procedure of the RSEC protocol for the continuous variables of the CV-QKD system.

Consequently, to reduce the BER of the slice, we could execute a random orthogonal rotation on the raw data before the slice quantization and then infer the last slice $S_{m}\left(x\right)$ according to multidimensional reconciliation. After decoding the *m*-th slice, Alice corrects the remaining slices in order. Assuming that Alice and Bob agree on the quantization function S(x) and the dimension *d* of the orthogonal matrix, the procedure of our improved protocol for reverse reconciliation is shown in Figure 2. The detailed process is described as follows:

***Step 1***: Alice and Bob divide their raw data into *d*-dimensional vectors as $X={\left\{x_{i}\right\}}^{d}$, $Y={\left\{y_{i}\right\}}^{d}$. Bob randomly generates a bit string $B={\left\{b_{i}\right\}}^{d}$ and chooses a point $U={\left\{\mu_{i}\right\}}^{d}$ on the unit sphere $O^{d-1}$ adjacent to the point $U_{B}={\left\{\frac{{\left(-1\right)}^{b_{i}}}{\sqrt{d}}\right\}}^{d}$. Then, he calculates an orthogonal matrix *M* satisfying MT=U for rotating *Y* to $Y^{\prime }=MY$, and informs Alice of the matrix *M*, where $T={\left\{t_{i}\right\}}^{d}$, $t_{i}=\frac{y_{i}}{∥Y∥}$.

***Step 2***: After receiving Bob’s orthogonal matrix *M*, Alice performs the same rotation on *X* and has the rotated data $X^{\prime }=MX$.

***Step 3***: Bob quantizes his rotated data $Y^{\prime }$ into *m*-slice bit vectors with the quantization function S(x), such as Equation (1), and sends the quantized slice values of the 1∼(l−1) slices $Q_{1},\cdots ,Q_{l-1}$ to Alice, where $Q_{i}=S_{i}\left(Y^{\prime }\right)$.

***Step 4***: Alice constructs a bit string ${\hat{Q}}_{m}$ of the *m*-th slice $Q_{m}$ from her rotated data $X^{\prime }$ using the slice estimator $\hat{S}$ as Equation (11) in Section 2.3. Subsequently, Bob uses a chosen error correction codes to generate a syndrome $S_{m}$ so that Alice aligns her bit string ${\hat{Q}}_{m}$ on the sequence $Q_{m}$.

***Step 5***: For each subsequent slice *k*, l≤k<m, Alice constructs a new string by applying the slice estimator $\hat{S}$ to $X^{\prime }$, and taking into account the disclosed slices $Q_{1},\cdots ,Q_{l-1}$ and the previously corrected bit strings $Q_{l},\cdots ,Q_{k-1},$$Q_{m}$. Again, Alice aligns her bit string to Bob’s sequence $Q_{k}$ using their chosen error correction codes and corresponding syndrome $S_{k}$.

### 2.3. Slice Estimator of RSEC

In the decoding stage of the RSEC reconciliation, we need to use the side information to estimate Bob’s quantized slices first. Let us now detail the expressions we proposed. According to the decoding process, we first estimate the last slice *m* of Bob. As is known,

(2) $$\begin{array}{c} Y^{\prime }-X^{\prime }=MY-MX\\ =MZ. \end{array}$$

where $M={\left(m_{ij}\right)}_{d\times d}$ is the rotation matrix, and $Z={\left\{z_{i}\right\}}^{d}$ follows the Gaussian distribution, $z_{i}∼N\left(0,\sigma^{2}\right)$.

As Gaussian variables have linear translation invariance—i.e., the linear combination of the independent Gaussian variables is still a Gaussian random variable—then $z_{i}^{\prime }=y_{i}^{\prime }-x_{i}^{\prime }$ follows the following Gaussian distribution

(3) $$z_{i}^{\prime }∼N\left(0,\sum_{j=1}^{d}m_{ij}^{2}\sigma^{2}\right).$$

It is known that $\sum_{j=1}^{d}m_{ij}^{2}=1,\left(i=1,2,\cdots ,d\right)$ since *M* is an orthogonal matrix. Therefore, the random variable $Z^{\prime }=Y^{\prime }-X^{\prime }$ has the same probability distribution as *Z*, i.e.,

(4) $$Y^{\prime }-X^{\prime }∼N{\left(0,\sigma^{2}\right)}^{d},$$

where $Z^{\prime }={\left\{z_{i}^{\prime }\right\}}^{d}$. Similarly, Bob’s rotated data $Y^{\prime }=MY$ follows the distribution $Y^{\prime }∼N{\left(0,\delta^{2}+\sigma^{2}\right)}^{d}$, and $X^{\prime }=MX$ follows the distribution $X^{\prime }∼N{\left(0,\delta^{2}\right)}^{d}$. In addition, according to the characteristics of the quantization function S(x), the bit string $Q_{m}=\left(Q_{m}^{1},Q_{m}^{2},\cdots \right)$ corresponds to the sign of the rotated data $Y^{\prime }$, i.e., if $y_{i}^{\prime }\geq 0$, $Q_{m}^{i}=1$, else, $Q_{m}^{i}=0,\;i=1,2,\cdots$. Here, we use $Q_{k}^{j}$ to denote the *k*-th slice of $y_{j}^{\prime }$. Hence, we obtain the conditional probability of $Q_{m}^{i}$ as follows

(5) $$P_{m}\left(Q_{m}^{i}|x_{i}^{\prime }\right)=\frac{K}{\sqrt{2\pi \sigma^{2}}}e^{\frac{-{\left(J\left(Q_{m}^{i}\right)\left|y_{i}^{\prime }\right|\;-\;x_{i}^{\prime }\right)}^{2}}{2\sigma^{2}}},$$

where $J\left(x\right)={\left(-1\right)}^{x+1}$, *K* is the normalization factor $P_{m}\left(Q_{m}^{i}=0|x_{i}\right)+P_{m}\left(Q_{m}^{i}=1|x_{i}\right)=1$. By integrating the conditional probability into a parameter, we find the soft information called the log likelihood ratio (LLR), which is a very useful parameter for estimation, as follows

(6) $$\ln\frac{P_{m}\left(Q_{m}^{i}=0|x_{i}^{\prime }\right)}{P_{m}\left(Q_{m}^{i}=1|x_{i}^{\prime }\right)}=-\frac{2x_{i}^{\prime }\left|y_{i}^{\prime }\right|}{\sigma^{2}}.$$

Given the transformation characteristics of the orthogonal rotation process, it is not difficult to deduce $y_{i}^{\prime }=\mu_{i}\left||Y|\right|$. If estimating $Q_{m}$ with Equation (6), Alice needs Bob to send his norm information ||Y||, which will lead to heavy communication traffic and storage resource requirements. Fortunately, we proposed a method that calculates the LLR without using the norm information of an encoder in our previous work [40]. Therefore, our protocol uses this improved method to calculate the LLR of $Q_{m}$ as follows

(7) $$LLR\left(Q_{m}^{i}\right)=S_{nr}||X||\ln\frac{1-v(x_{i}^{\prime })}{1+v(x_{i}^{\prime })},$$

where $S_{nr}$ is the SNR of the quantum channel, and $v\left(x_{i}^{\prime }\right)=\frac{x_{i}^{\prime }}{\left||X|\right|}$.

For the remaining slices *k* (l≤k<m), we derive their LLR with the corrected slices and the received l−1 slices as prior information. According to the previous analysis, we find the joint density function of the rotated data $X^{\prime }$ and $Y^{\prime }$ as Equation (8). Hence, the random variables $X^{\prime }$ and $Y^{\prime }$ follow the joint density function,

(8) fX′Y′(x,y)=12πδσe−x2x22δ22δ2e−(y−x)2(y−x)22σ22σ2.

According to Equation (8) and the characteristics of the quantization function Equation (1), we derive that the conditional probability of $Q_{k}^{i}$ is expressed as

(9) $$P_{k}\left(Q_{k}^{i}=b|x_{i}^{\prime },Q_{1,\cdots ,k-1,m}^{i}\right)=\sum_{\tau }\int_{\tau_{a-1}}^{\tau_{a}}f_{X^{\prime }Y^{\prime }}\left(x_{i}^{\prime },y\right)dy,$$

where τ represents those quantization intervals satisfying $S_{1,\cdots ,k-1}\left(y\right)=B$, $S_{k}\left(y\right)=b$, i.e., $\tau =\{\left(\tau_{a-1},\tau_{a}\right)|\forall y\in \left(\tau_{a-1},\tau_{a}\right),S_{1,\cdots ,k-1,m}\left(y\right)=B,S_{k}\left(y\right)=b\}$, b= 0 or 1, $B=(Q_{1,\cdots ,k-1}^{i},Q_{m}^{i})$ denotes the disclosed and corrected slices.

Accordingly, we find the initial LLR of $Q_{k}^{i}$ as Equation (10) to preliminarily estimate the rotated results of the *k*-th slice,

(10) LLR(Qki)=ln∑τ0∫τa−1τae−x′i2x′i22δ22δ2e−(y−xi′)2(y−xi)22σ22σ2dy∑τ1∫τa′−1τa′e−x′i2x′i22δ22δ2e−(y−xi′)2(y−xi′)22σ22σ2dy,l≤k<m,

where $\tau^{0}$ represents the quantization intervals that satisfy $S_{1,\cdots ,k-1,m}\left(y\right)=B,S_{k}\left(y\right)=0$, and $\tau^{1}$ satisfies $S_{1,\cdots ,k-1,m}\left(y\right)=B,S_{k}\left(y\right)=1$, respectively.

Based on the derived LLRs of each slice, the estimator $\hat{S}$ of our RSEC reconciliation is constructed as follows

(11) $$\hat{S}\left(Q_{j}^{i}\right)=\;\left\{\begin{array}{cc} \; & 0\;,\mathrm{if}\;LLR(Q_{j}^{i})>0\\ & \;\;\;1\;,\mathrm{otherwise} \end{array}\right..$$

Then, Alice can use Equation (11) to construct an initial estimation ${\hat{Q}}_{j}^{i}=\hat{S}\left(Q_{j}^{i}\right)$ for Bob’s slice value $Q_{j}^{i}$.

### 2.4. Error Probability of Slice Estimator

In order to evaluate the accuracy of the proposed slice estimator, we theoretically analyze and compare the error probability of the SEC protocol and the RSEC protocol. The error probability of the slice estimator denotes the theoretical probability that Alice’s slice estimator yields a result different from Bob’s slice. For the SEC protocol, the error probability in slice *i* (l≤i≤m) can be expressed as

(12) $$\begin{array}{c} e_{i}^{w}=P\left[S_{i}\left(Y\right)\neq {\hat{S}}_{i}^{w}\left(X\right)\right]\\ \;=\sum_{D_{b_{1}\cdots b_{i-1}}}\left(P\left[S_{i}\left(Y\right)=0\right]\;\cdot \;P\left[{\hat{S}}_{i}^{w}\left(X\right)=1|S_{1\cdots i-1}\left(Y\right)\right]+\right.\\ \left.\;\;\;P\left[S_{i}\left(Y\right)=1\right]\;\cdot \;P\left[{\hat{S}}_{i}^{w}\left(X\right)=0|S_{1\cdots i-1}\left(Y\right)\right]\right), \end{array}$$

where $b_{1}\cdots b_{i-1}\in GF{\left(2\right)}^{i-1}$ and $D_{b_{1}\cdots b_{i-1}}=\;\left\{t|S_{1\cdots i-1}\left(t\right)=b_{1}\cdots b_{i-1}\right\}$, the superscript *w* characterize the variables of the SEC protocol. As the random variables *X* and *Y* follow the Gaussian distribution symmetrical about the coordinate axis, it is easy to obtain that $P\left[S_{i}\left(Y\right)=0\right]\;\cdot \;P\left[{\hat{S}}_{i}^{w}\left(X,S_{1\cdots i-1}\left(Y\right)\right)=1\right]=P\left[S_{i}\left(Y\right)=1\right]\;\cdot \;P\left[{\hat{S}}_{i}^{w}\left(X,S_{1\cdots i-1}\left(Y\right)\right)=0\right]$, and then $e_{i}^{w}$ can be further written as

(13) $$e_{i}^{w}=2\sum_{D_{b_{1}\cdots b_{i-1}}}P\left[S_{i}\left(Y\right)=0\right]\;\cdot \;P\left[{\hat{S}}_{i}^{w}\left(X\right)=1|S_{1\cdots i-1}\left(Y\right)\right],$$

each of these terms can be expanded as

$$P\left[S_{i}\left(Y\right)=0\right]\;=\int_{A_{b_{1}\cdots b_{i-1}0}}\frac{1}{\sqrt{2\pi (\delta^{2}+\sigma^{2})}}e^{\frac{-y^{2}}{2(\delta^{2}+\sigma^{2})}}dy,$$

$$P\left[{\hat{S}}_{i}^{w}\left(X\right)=1|S_{1\cdots i-1}\left(Y\right)\right]=\int_{B_{b_{1}\cdots b_{i-1}}}\frac{1}{\sqrt{2\pi \delta^{2}}}e^{\frac{-x^{2}}{2\delta^{2}}}dx,$$

where $B_{b_{1}\cdots b_{i-1}}=\left\{x|\int_{A_{b_{1}\cdots b_{i-1}1}}f_{XY}\left(x,y\right)dy>\int_{A_{b_{1}\cdots b_{i-1}0}}f_{XY}\left(x,y\right)dy\right\}$ denotes the set in which ${\hat{S}}_{i}^{w}\left(x\right)=1$ has a higher probability than ${\hat{S}}_{i}^{w}\left(x\right)=0$, and $f_{XY}\left(x,y\right)=\frac{1}{2\pi \delta \sigma }e^{-\frac{x^{2}}{2\delta^{2}}-\frac{{\left(y-x\right)}^{2}}{2\sigma^{2}}}$, $A_{b_{1}\cdots b_{i-1}a}=\;\left\{y|S_{1\cdots i-1}\left(y\right)=b_{1}\cdots b_{i-1}\wedge S_{i}\left(y\right)=a\right\}$.

In the RSEC protocol, the quantized values in slice *m* are estimated and decoded first. According to the characteristics of the quantization function S, Bob’s last slice $Q_{m}=0$ when y<0 and $Q_{m}=1$ otherwise. On the other side, Alice constructs an estimation of Bob’s quantized value $Q_{m}$ with Equations (7) and (11). The result in the last slice yielded by our estimator $\hat{S}\left(x\right)$ corresponds to the sign of *x*: ${\hat{Q}}_{m}=0$ when x<0 and ${\hat{Q}}_{m}=1$ otherwise. Therefore, the error probability of the RSEC protocol in the last slice *m* can be expressed as

(14) $$\begin{array}{c} e_{m}^{p}=P\left[S_{m}\left(Y^{\prime }\right)\neq {\hat{S}}_{m}\left(X^{\prime }\right)\right]\\ =P\left(Y^{\prime }>0\right)\;\cdot \;P\left(X^{\prime }<0|Y^{\prime }>0\right)\;+\;P\left(Y^{\prime }<0\right)\;\cdot \;P\left(X^{\prime }>0|Y^{\prime }<0\right), \end{array}$$

the superscript *p* characterize the variables of the RSEC protocol. By applying the probability distribution of $X^{\prime }$ and $Y^{\prime }$ as analyzed in the previous section, we have $P\left(Y^{\prime }>0\right)\;=\;P\left(Y^{\prime }<0\right)\;=\frac{1}{2}$, and the $e_{m}^{p}$ can be further calculated as follows

(15) emp=12∫−∞012πδe−x2x22δ22δ2dx∫0+∞12πσe−(y−x)2(y−x)22δ22δ2dy+12∫0+∞12πδe−x2x22δ22δ2dx∫−∞012πσe−(y−x)2(y−x)22δ22δ2dy=∫0+∞∫−∞0fX′Y′(x,y)dxdy,

where $f_{X^{\prime }Y^{\prime }}\left(x,y\right)$ is given in Equation (8), and the second equation in Equation (15) is transformed by using the symmetry of the probability distribution of $X^{\prime }$ and $Y^{\prime }$.

Similar to the SEC protocol, the quantized values of the RSEC protocol in other slice *k* (l≤k≤m−1) are estimated by using the disclosed slices and the successfully decoded slices. Thus, the error probability of these slices can be calculated according to Equation (13), where the last slice $S_{m}\left(Y\right)$ is taken as additional information.

**Figure 3:**
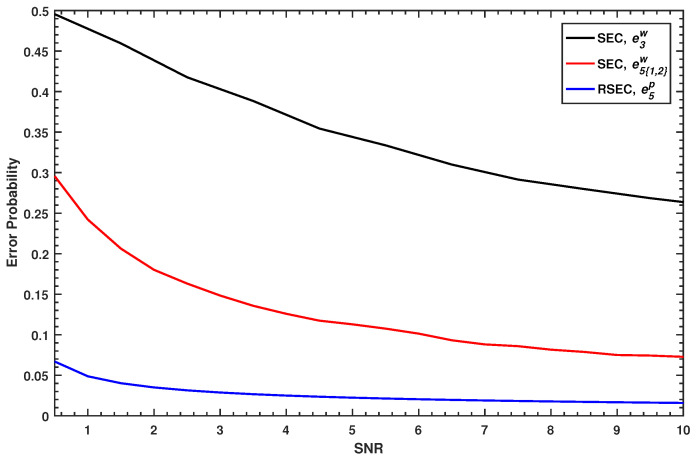
Comparison of the error probability between the RSEC and the SEC protocol at different SNRs.

The error probabilities of the subsequent slices—which recursively depend on that of all the previous slices—are not simple to calculate [22]. Here, we compare the estimation accuracy of RSEC protocol with the SEC protocol only by the error probability in the first decoded slice (i.e., the *l*-th slice in SEC, the *m*-th slice in RSEC). Using the Equations (13) and (15), we find the error probability in slice l=3 of the SEC protocol and the error probability in slice m=5 of RSEC protocol as shown in Figure 3, where the first two slices are disclosed and the quantization adopts the five-slices function.

$e_{5}^{p}$ corresponding to the blue curve is always smaller than $e_{3}^{w}$ corresponding to the black curve, i.e., the estimator of RSEC in the first decoded slice always has a lower error probability than that of SEC. If the *m*-th slice in the SEC protocol is estimated first, its error probability $e_{5\{1,2\}}^{w}$ corresponding to the red curve, of which the expression can be given by Equation (16), also always performs worse than that of the RSEC protocol.

(16) $$e_{5\{1,2\}}^{w}=2\sum_{D_{b_{1}b_{2}}}P\left[S_{5}\left(Y\right)=0\right]\;\cdot \;P\left[{\hat{S}}_{5}^{w}\left(X\right)=1|S_{1,2}\left(Y\right)\right].$$

From the above analysis, it is indicated that the proposed estimator of RSEC protocol has much higher estimation accuracy in the quantization phase than the original estimator of the SEC protocol. Note that the error probability represents the theoretical case of the bit error rate, and the bit error rate of each slice needs to be estimated separately in the practical reconciliation.

### 2.5. Reconciliation Efficiency

Let us now discuss the reconciliation efficiency of the proposed protocol, which is an important indicator for evaluating the performance of the reconciliation procedure. As is known, the random orthogonal rotation operation on raw data does not expose any information of the rotated results [24]. According to the definition of efficiency in the slice reconciliation [33], the reconciliation efficiency β of the RSEC protocol can be expressed as

(17) $$\beta =\frac{H\left(S\left(Y^{\prime }\right)\right)-m+\sum_{i=1}^{m}R_{i}}{I(X,Y)},$$

where $I\left(X;Y\right)=\frac{1}{2}\log_{2}\left(1+S_{nr}\right)$ is the classical capacity of the quantum channel for Gaussian variables, *m* denotes the number of slices of quantization function, and $R_{i}$ represents the code rate of the error correction scheme of the *i*-th slice. $H(S\left(Y^{\prime }\right))$ is the entropy of the slice sequences $S(Y^{\prime })$, which can be calculated as follows

(18) $$H\left(S\left(Y^{\prime }\right)\right)=-\sum_{a}P_{a}\log_{2}P_{a},$$

with

(19) $$P_{a}=\frac{1}{2}\left(erf\left(\frac{\tau_{a}}{\sqrt{2(\delta^{2}+\sigma^{2})}}\right)\;-\;erf\left(\frac{\tau_{a-1}}{\sqrt{2(\delta^{2}+\sigma^{2})}}\right)\right),$$

where $\tau_{a}$ denotes the point dividing the real numbers R, $1\leq a\leq 2^{m}$, and $\tau_{0}=-\infty$, $\tau_{2^{m}}=+\infty$. $\delta^{2}$ and $\sigma^{2}$ represent Alice’s modulation variance and the noise variance, respectively.

Generally, the code rate of the first l−1 slices are equal to 0 since they are disclosed via the authentic classical channel.

## 3. Implementation of RSEC with Polar Codes

After quantizing the continuous variables into strings of bits with slice functions, the legitimate parties are needed to further apply a classical error correction code to complete the reconciliation of the correlated raw data. In this section, we will implement the RSEC protocol with polar codes to distill the correct keys from the correlating raw data.

### 3.1. Review of Polar Codes

The polar code is an error correction code that has been strictly proven to achieve the Shannon capacity [48]. The recursive structure of its encoding and decoding gives them good practical performance. This is relatively easier to construct than another commonly used code, i.e., LDPC code. Therefore, we chose polar codes to implement the RSEC protocol in this research. The RSEC can also be implemented with other error correction codes. Now, we briefly review the encoding and decoding of polar codes in traditional communication.

#### 3.1.1. Encoding

The central idea of polar codes is to convert the *N* individual copies of the channel *W* into two different types of channels, i.e., error-free channel and completely noisy channel, through an operation called channel polarization—channel combining and channel splitting. The information sender chooses the positions corresponding to the error-free channel to place her message bits (called information bits), and usually sets the remaining positions corresponding to the completely noisy channel as 0 (called frozen bits). The information bits and frozen bits together form a sequence $u_{1}^{N}$ of *N* bits. We use the notation $u_{1}^{n}=\left(u_{1},\cdots ,u_{n}\right)$ to denote a row vector of *n* bits. The sender encodes the sequence $u_{1}^{N}$ to a codeword $x_{1}^{N}$ by

(20) $$x_{1}^{N}=u_{1}^{N}G,$$

where *G* is the generator matrix and defined as $G=F^{⊗\log_{2}N}B$, $F^{⊗n}$ means to perform the Kronecker product *n* times on the matrix $F\overset{\Delta }{\;=}\left[\begin{array}{cc} 1 & 0\\ 1 & 1 \end{array}\right]$, and *B* is a permutation matrix for executing the bit-reversal operation [48]. Obtaining the codeword $x_{1}^{N}$, the sender transmits it to the information receiver for decoding.

#### 3.1.2. Decoding

After the codeword $x_{1}^{N}$ is transmitted through the channel, the receiver obtains a sequence $y_{1}^{N}$, which is a noise version of $x_{1}^{N}$. Then, he uses successive cancellation (SC) or successive cancellation list (SCL) decoding algorithms to correct the error bits among $y_{1}^{N}$ with the given frozen bits. We here describe the receiver’s decoding process with a SC decoding algorithm [48]:

1. Initialize the received information $y_{1}^{N}$ with channel transition probability W(y|x) as
  (21) $$L_{1}^{\left(j\right)}\left(y_{j}\right)=\frac{W(y_{j}|0)}{W(y_{j}|1)},\;\;j=1,2,\cdots ,N.$$
2. Calculate the likelihood ratio (LR) of $u_{j}$ with the decoding results ${\hat{u}}_{1}^{j-1}=\;({\hat{u}}_{1},{\hat{u}}_{2},$$\cdots ,{\hat{u}}_{j-1})$ of the previous j−1 bits as follows
  (22) $$L_{N}^{\left(j\right)}\left(y_{j},{\hat{u}}_{1}^{j-1}\right)\;=\frac{W_{N}^{\left(j\right)}\left(y_{1}^{N},{\hat{u}}_{1}^{j-1}|u_{j}=0\right)}{W_{N}^{\left(j\right)}\left(y_{1}^{N},{\hat{u}}_{1}^{j-1}|u_{j}=1\right)},$$
  where
  (23) $$W_{N}^{\left(j\right)}\left(y_{1}^{N},u_{1}^{j-1}|u_{j}\right)\overset{\Delta }{\;=}\frac{1}{2^{N-1}}\sum_{u_{j+1}^{N}\;\in \;{\left\{0,1\right\}}^{N-j}}W_{N}\left(y_{1}^{N}|u_{1}^{N}\right),$$
  and
  (24) $$W_{N}\left(y_{1}^{N}|u_{1}^{N}\right)\;=W^{N}\left(y_{1}^{N}|x_{1}^{N}=u_{1}^{N}G\right)\;=\prod_{i=1}^{N}W\left(y_{i}|x_{i}\right).$$
3. Generate the decision ${\hat{u}}_{j}$ of $u_{j}$ as
  (25) $${\hat{u}}_{j}=\;\left\{\begin{array}{cc} \; & u_{j}\;\;,\mathrm{if}\;j\in A\\ \; & 0\;\;\;,\mathrm{if}\;j\notin A\;\mathrm{and}\;L_{N}^{\left(j\right)}\left(y_{1}^{N},{\hat{u}}_{1}^{j-1}\right)\;\geq \;1\\ \; & 1\;\;\;,\mathrm{if}\;j\notin A\;\mathrm{and}\;L_{N}^{\left(j\right)}\left(y_{1}^{N},{\hat{u}}_{1}^{j-1}\right)\;<\;1 \end{array}\right.,$$
  where A is the position set of the frozen bits.

After getting the *j*-th bit by step (iii), the process returns to step (ii) to decode the (j+1)-th bit.

### 3.2. Implementation Process

The reconciliation mode of CV-QKD is different from traditional communication. In traditional communication, the codeword is mixed with noises during the reconciliation. However, in a CV-QKD system, the two parties have already shared inconsistent data before the post-processing phase, in other words, the noise in the codeword appeared before the reconciliation. Therefore, in order to correct the slice sequences of RSEC, it is necessary to establish a virtual channel for Alice and Bob to deal with the noise.

The encoding of polar codes is reversible: Encoding an input sequence *x* twice, one can recover this sequence, i.e., xGG=x. This property can be used to establish a virtual channel as: Bob encodes a slice sequence *x* to another sequence u=xG, and then sends the bits $u_{A}$ corresponding to the frozen indices to Alice. Since uG=(xG)G=x, the slice sequence *x* can be regarded as a polar codeword, Alice’s initial estimation $\hat{S}\left(x\right)$ of Bob’s slice value can be viewed as the received codeword, and $u_{A}$ corresponds to the frozen bits shared by the two parties. Therefore, a virtual channel can be established by using the above method.

**Figure 4:**
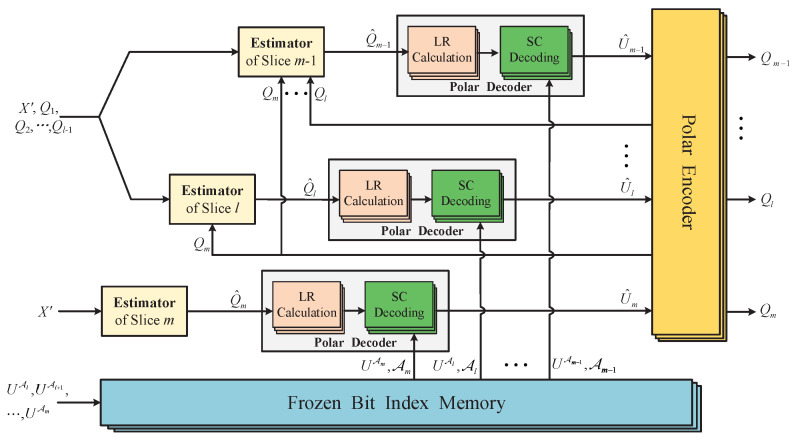
Logic structure of the RSEC reconciliation with polar codes. The LR calculation modules provide the initial LR $L_{1,i}^{\left(j\right)}\left({\hat{q}}_{j}\right)$, and the frozen bit index memory stores the frozen bit $U^{A_{i}}$ and frozen index set $A_{i}$.

Before launching the reconciliation with polar codes, Alice and Bob determine the code rate $R_{i}^{\prime }$ of each slice according to the SNR and share the corresponding frozen index set $A_{i}$. The frozen index set can be selected by a construction algorithm with consideration to $R_{i}^{\prime }$. Then, the logic structure of the RSEC reconciliation with polar codes is shown in Figure 4, in which the detailed implementation process is described as follows:

***Step 1***: Alice and Bob convert their correlated data *X*, *Y* to another continuous-variable sequence noted as $X^{\prime }$, $Y^{\prime }$ with random orthogonal rotation according to RSEC. Bob then quantizes $Y^{\prime }$ into *m* slice sequences $Q_{1},Q_{2},\cdots ,Q_{m}$ with the slice function and sends the first l−1 slices $Q_{1},Q_{2},\cdots ,Q_{l-1}$ to Alice. Afterward, they begin to reconcile the remaining slice sequences with polar codes in the order of m,l,l+1,⋯,m−1 slice.

***Step 2***: Alice uses the proposed estimator in Equation (11) to construct a bit string ${\hat{Q}}_{i}$ corresponding to Bob’s slice sequence $Q_{i}$. Meanwhile, Bob encodes his slice sequence to $U=Q_{i}G$, and sends the bits $U^{A_{i}}$ at the frozen positions to Alice.

***Step 3***: Alice calculates the initial LR $L_{1,i}^{\left(j\right)}\left({\hat{q}}_{j}\right)$ as Equation (26), j=1,2,⋯,N, and then makes a decision $\hat{U}$ on *U* after getting the final LR $L_{N,i}^{\left(j\right)}\left({\hat{q}}_{1}^{N},{\hat{u}}_{1}^{j-1}\right)$ in Equation (27). Afterward, she can recover Bob’s sequence $Q_{i}$ with a high probability by executing an encoding operation on $\hat{U}$.

(26) $$L_{1,i}^{\left(j\right)}\left({\hat{q}}_{j}\right)=\frac{W({\hat{q}}_{j}|0)}{W({\hat{q}}_{j}|1)},\;\;j=1,2,\cdots ,N,$$

where $L_{1,i}^{\left(j\right)}\left({\hat{q}}_{j}\right)$ is the initial LR corresponding to the *j*-th bit of *U*, ${\hat{q}}_{j}$ is the *j*-th bit of ${\hat{Q}}_{i}$, $U=(u_{1},\cdots ,u_{N})$, $\hat{U}=\left({\hat{u}}_{1},\cdots ,{\hat{u}}_{N}\right)$, the channel transition probability can be calculated as: if y=x, $W\left(y|x\right)=1-e_{i}$, if y≠x, $W\left(y|x\right)=e_{i}$. The bit error rate $e_{i}$ can be estimated in the stage of parameter estimation by executing the quantization operation on the extra raw data.

(27) $$L_{N,i}^{\left(j\right)}\left({\hat{q}}_{1}^{N},{\hat{u}}_{1}^{j-1}\right)=\frac{W_{N}^{\left(j\right)}\left({\hat{q}}_{1}^{N},{\hat{u}}_{1}^{j-1}|0\right)}{W_{N}^{\left(j\right)}\left(x_{1}^{{}^{\prime }N},{\hat{u}}_{1}^{j-1}|1\right)}.$$

Moreover, Equation (27) can evolve in a recursive manner as

if *j* is odd, i.e., j=2k−1, then
  (28) $$L_{N,i}^{\left(2k-1\right)}\left({\hat{q}}_{1}^{N},{\hat{u}}_{1}^{2k-2}\right)=f\left(L_{N/2,i}^{\left(k\right)}\left({\hat{q}}_{1}^{N/2},{\hat{u}}_{1,o}^{2k-2}⊕{\hat{u}}_{1,e}^{2k-2}\right),L_{N/2,i}^{\left(k\right)}\left({\hat{q}}_{N/2+1}^{N},{\hat{u}}_{1,e}^{2k-2}\right)\right),$$
if *j* is even, i.e., j=2k, then
  (29) $$L_{N,i}^{\left(2k\right)}\left({\hat{u}}_{1}^{N},{\hat{u}}_{1}^{2k-1}\right)=g\left(L_{N/2,i}^{\left(j\right)}\left({\hat{q}}_{1}^{N/2},{\hat{u}}_{1,o}^{2k-2}⊕{\hat{u}}_{1,e}^{2k-2}\right),L_{N/2,i}^{\left(k\right)}\left({\hat{q}}_{N/2+1}^{N},{\hat{u}}_{1,e}^{2k-2}\right),{\hat{u}}_{2k-1}\right),$$
  where $f\left(a,b\right)=\frac{a\cdot b+1}{a+b}$, $g\left(a,b,s\right)=a^{1-2s}\cdot b$, we use $x_{a,o}^{b}$ to denote the odd terms of $x_{a}^{b}$, and $x_{a,e}^{b}$ denotes the even terms of $x_{a}^{b}$.

Alice can also use LLR as the soft information of polar codes for decoding. In this case, the initial LLR is calculated according to Equations (7) and (10).

In order to ensure that the equation $\hat{U}G=Q_{i}$ holds with a high probability, Alice and Bob need to perform a cyclic redundancy check (CRC) to verify the decoding result $\hat{U}$. If $\hat{U}$ fails to pass CRC check, Alice and Bob give up on this slice $Q_{i}$. Even if the decoding result passes the CRC check, undetected error bits may still exist. However, this situation rarely occurs and can be overlooked.

As the CRC values will leak the information about $Q_{i}$, it is necessary to discard them. Therefore, the code rate $R_{i}$ of each slice is calculated as follows

(30) $$R_{i}=R_{i}^{\prime }-\frac{n_{crc}}{N},$$

where $n_{crc}$ is the length of the CRC values.

## 4. Experiment Results and Analysis

To evaluate the performance of the RSEC protocol, we performed a series of experiments to compare their performances, including the quantization efficiency, reconciliation efficiency, and the secret key rate.

### 4.1. Quantization Efficiency of RSEC

The principle of quantization is to minimize the information loss so that $I(X^{\prime };S\left(Y^{\prime }\right))$ can be made arbitrarily close to the initially shared information I(X;Y). After quantization, the mutual information $I(X^{\prime };S\left(Y^{\prime }\right))$ shared by Alice and Bob can be expressed as

(31) $$I\left(X^{\prime };S\left(Y^{\prime }\right)\right)=H\left(S\left(Y^{\prime }\right)\right)-\left[H\left(S_{m}\left(Y^{\prime }\right)|X^{\prime }\right)+\sum_{i=1}^{m-1}H(S_{i}\left(Y^{\prime }\right)|X^{\prime },S_{1\ldots i-1,m}\left(Y^{\prime }\right))\right],$$

where $S\left(Y^{\prime }\right)=\left(S_{1}\left(Y^{\prime }\right),\cdots ,S_{m}\left(Y^{\prime }\right)\right)$ are the slice values of Bob.

As the conditional entropy of Equation (31) recursively depends on all previously estimated results, calculating $I(X^{\prime };S\left(Y^{\prime }\right))$ is not a simple task. For this reason, it is common practice to replace the conditional entropy with $H(e_{i})$ equivalently [22]. Then, the goal of quantization is simply to minimize each $e_{i}$, of which $H(e_{i})$ is an increasing function for $0\leq e_{i}<0.5$, $e_{i}$ is the BER of *i*-th slice. Therefore, the quantization efficiency $\beta_{s}$ can be measured with Equation (32) equivalently [22],

(32) $$\beta_{s}=\frac{H\left(S\left(Y^{\prime }\right)\right)-H_{e}}{I(X;Y)},$$

with $H_{e}=\sum_{i=1}^{m}H(e_{i})$, $H\left(e_{i}\right)=-e_{i}log_{2}\left(e_{i}\right)-\left(1-e_{i}\right)log_{2}\left(1-e_{i}\right)$.

**Figure 5:**
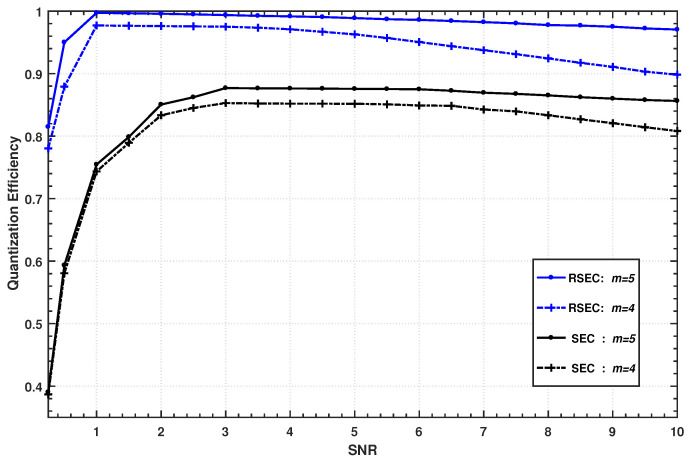
The quantization efficiency of the RSEC and the SEC protocol with four and five slices at different SNR. The upper two curves denote the values of the RSEC protocol; the lower two curves correspond to the values of the SEC protocol.

Figure 5 shows the quantization efficiency curves of SEC and RSEC at different SNR when m=4 and m=5. As can be seen from the figure, the quantization efficiency of SEC drops sharply for SNR <3, which confirms that SEC reconciliation usually performs poorly at low SNRs. By contrast, RSEC can still maintain a high quantization efficiency $\beta_{s}>96\%$ for almost all SNRs <3, and even achieves above 99% quantization efficiency in the range of SNR ∈(1,3) when adopting the five-slice scheme. The primary reason for the better performance of the proposed RSEC over the SEC protocol is attributed to our new estimator.

With the orthogonal rotation, our estimator can estimate Bob’s slice sequences more accurately, especially for the slice that is decoded first, and thus the error rate $e_{i}$ decreases accordingly. Moreover, the result in Figure 5 also confirms the following basic facts. For a fixed SNR, the higher the number of slices, the lower the information loss caused by quantization.

### 4.2. Reconciliation Efficiency of RSEC with Polar Codes

It appears that the polarization speed of polar codes is highly dependent on the channel [49]. Compared with the Binary Input Additive White Gaussian Noise Channel (BIAWGNC), constructing polar code for a Binary Symmetric Channel (BSC) is relatively uncomplicated and more common. Moreover, a BSC can be established between the two parties if Alice makes an initial estimation of Bob’s slice sequences using LLR values. Accordingly, in our experiments, we construct the polar codes on a BSC and calculate the initial LR as Equation (26) for decoding the slice sequences.

**Figure 6:**
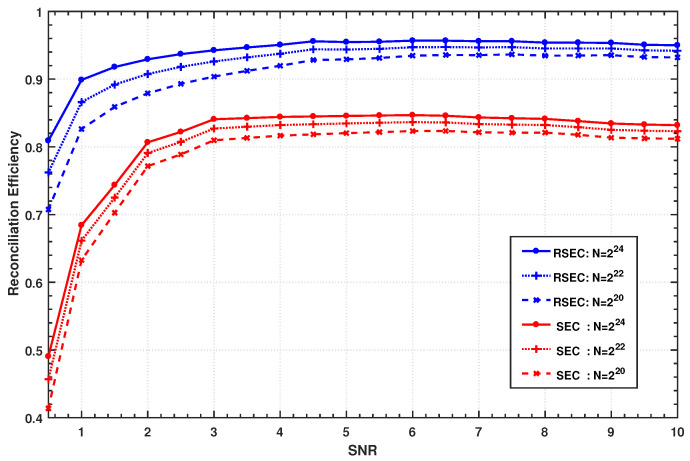
The reconciliation efficiency of the RSEC and SEC protocol with polar codes at different SNRs when the number of slices m=5. The upper three curves show the values of the RSEC protocol; the lower three curves correspond to the values of the SEC protocol. For the RSEC and SEC protocols, their three curves from bottom to top represent the reconciliation efficiencies obtained with $N=2^{20}$, $2^{22}$, $2^{24}$, respectively.

Figure 6 compares the reconciliation efficiencies of the RSEC and the SEC protocol with polar codes when m=5. The 32-bit CRC is adopted for polar codes to check the decoding results, i.e., $n_{crc}=32$, and the eight-dimensional orthogonal matrix is used in rotation. For a fixed SNR value and different block length, 1000 blocks of raw data are generated to measure the reconciliation performance. The experimental results are obtained with frame error rate (FER) ≤0.1, but a null BER in the blocks decoded successfully.

Combining Figure 5 and Figure 6, it is not difficult to find that the curvilinear trend of the quantization efficiency is essentially consistent with that of the reconciliation efficiency; this is because the reconciliation scheme with good quantization performance usually performs better in reconciliation. Hence, the reconciliation efficiency of the proposed protocol is higher than the SEC protocol over the entire range in Figure 6 due to its higher quantization efficiency.

As shown in Figure 6, both the reconciliation efficiencies of RSEC and SEC increase with the increasing block length of polar codes since the decoding performance of polar codes will become better with the increase of its block size. The proposed RSEC protocol has an efficiency above 90% over almost the entire range SNR ≥1 for the block lengths starting from $2^{24}$, and even exceeds 95% at SNR >3, which allows the system to distill more than 1 bit corrected key per raw data.

RSEC has a high quantization efficiency in the SNR range (1,3), whereas its reconciliation efficiency is not so perfect. The reason is that the relatively low SNR leads to a high BER >10% in some noisy slices, and the decoding performance of polar codes decreases at high BER [50]. In fact, the high quantization efficiency of RSEC allows the system to achieve a higher reconciliation efficiency by using a high-performance code.

In addition, we compare the reconciliation efficiency values with the representative works on SEC in Table 1. As shown in the table, the proposed protocol improves almost all previously published reconciliation efficiencies in terms of the SEC protocol in the high SNR regime, which is the main focus of the SEC reconciliation. In fact, the reconciliation efficiency values of [33] listed in the table are obtained under an optimistic situation of adopting the optimal number of slices and specially designed high-performance codes. Nevertheless, our reconciliation scheme still has a competitive advantage over [33] on the whole.

Many achievements have also been made in multidimensional reconciliation, for example, [38] implements eight-dimensional reconciliation with β=99% and FER =0.883 using QC MET-LDPC code at SNR =0.0283, and [39] achieves β=93.40%,95.84%,96.99% and FER ≤0.375 with eight-dimensional reconciliation based on the MET-LDPC code at SNRs of =0.160,0.075,0.029, respectively. However, unlike the SEC protocol, the multidimensional reconciliation protocol is more suitable for the low SNRs rather than the high SNR regime. The existing works on multidimensional reconciliation are aimed at the extremely low SNRs and rarely provide the experimental results in the high SNR regime. Therefore, we mainly give a comparison with the representative results of the SEC protocol.

### 4.3. Secret Key Rate of RSEC

Assuming a collective Gaussian attack and accounting for the finite-size effects, the secret key rate of a CV-QKD system with reverse reconciliation can be expressed as [29]:

(33) $$K_{finite}=\frac{N_{data}}{N_{total}}\left[\beta I_{AB}-\chi_{BE}-\Delta \left(N_{data}\right)\right],$$

where $N_{total}$ is the total number of symbols sent from Alice to Bob, $N_{data}$ is the number of raw data used for key distillation, β is the reconciliation efficiency, $I_{AB}$ denotes the mutual information between Alice and Bob, $\chi_{BE}$ denotes the Holevo bound on the information that Eve can obtain, and $\Delta (N_{data})$ is the finite-size offset factor. $I_{AB}$ and $\chi_{BE}$ are related to the physical parameters, including the transmittance *T*, the total noise $\chi_{total}$, and Alice’s modulation variance $V_{A}$. The transmittance *T* of the quantum channel is defined as T=10−αLαd1010, where α is the single-mode fiber transmission loss and L is the transmission distance.

The total noise $\chi_{\mathrm{total}}$ consists of the channel added noise and the noise generated by Bob’s detector, and be given by $\chi_{\mathrm{total}}=\chi_{\mathrm{line}}+\frac{\chi_{\mathrm{hom}}}{T}$, where $\chi_{\mathrm{line}}=\left(\frac{1}{T}-1\right)+\xi$ and $\chi_{\mathrm{hom}}=\frac{1+V_{el}}{\eta }-1$, ξ is the excess channel noise, $V_{el}$ denotes the added electronic noise of Bob’s detector, and η represents the detector efficiency. The detailed calculation about $I_{AB}$, $\chi_{BE}$, and $\Delta (N_{data})$ can be found in Appendix A.

In our simulation, the experimental physical parameters reported in previously published work [29] were used to characterize the CV-QKD system and quantum channel. Optimizing the modulation variance $V_{A}$ for each transmission distance can maximize the SNR of a quantum channel. The modulation variance $V_{A}$ in our work is adjusted according to [38]. We chose $N_{total}=2N_{data}$ and the security parameter of $10^{-10}$ for $\Delta (N_{data})$ [18].

**Figure 7:**
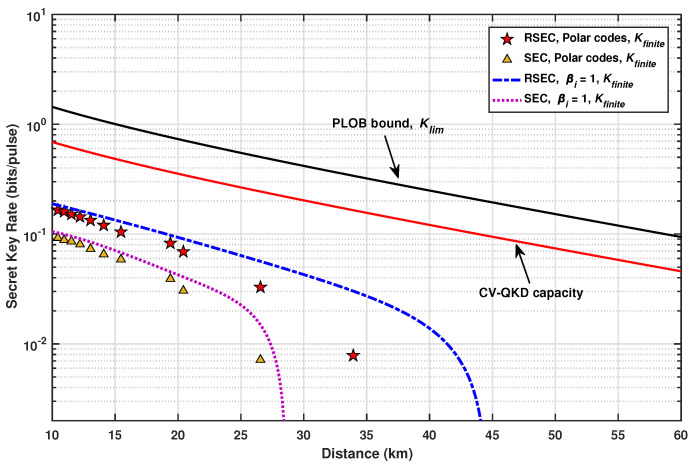
Finite secret key rate with $N_{total}=2^{40}$ vs. distance. The five-pointed stars correspond to the secret key rates using five-slice RSEC with polar codes of $N=2^{24}$; the triangle points correspond to the values using five-slice SEC with polar codes of $N=2^{24}$; the blue dotted line and purple dotted line represent the asymptotic theoretical secret key rates using five-slice RSEC and SEC with a perfect error correction scheme (i.e., $\beta_{i}=1$), respectively. Other parameters are as follows [29]: α=0.2dB/km, ξ=0.005, $V_{el}=0.041$, η=0.606.

Figure 7 presents the finite secret key rates $K_{finite}$ over the transmission distances with $N_{finite}=2^{40}$ bits. The five-pointed stars and triangle points compare the secret key rates achieved with polar codes of block lengths $N=2^{24}$ bits, where the CV-QKD system using RSEC always provides higher secret key rates than that using SEC at the same transmission distance. Using RSEC reconciliation, we achieve a secret key rate of $7.83\times 10^{-3}$ bits/pulse at a distance of 33.93 km, while the CV-QKD system using SEC cannot provide any secret key.

In particular, assuming perfect error correction in the decoding of each slice, the RSEC protocol has more obvious advantages than the SEC protocol in the asymptotic secret key rate as the two dotted lines in Figure 7. A perfect error correction scheme allows each slice to achieve its Shannon capacity, i.e., the efficiency $\beta_{i}$ of error correction in each slice is assumed as 1. With the increase of the transmission distance, the secret key rate of CV-QKD decreases. This is because the SNR becomes lower with the increase of transmission distance, which leads to the reduction of the quantification efficiency. Notably, when the transmission distance increases to about 30 km, the CV-QKD system using the SEC protocol can hardly generate any secret key. However, the RSEC protocol can theoretically extend the secure distance of the CV-QKD system to about 45 km.

There exists an upper bound called the Pirandola–Laurenza–Ottaviani–Banchi (PLOB) bound for the secret-key capacity of a lossy channel. The PLOB bound $K_{lim}$ is determined by the transmittance *T* of channel and is given by [51]

(34) $$K_{lim}=-log_{2}\left(1-T\right).$$

The black solid line in Figure 7 is the PLOB bound, which sets the fundamental rate limit for point-to-point QKD in the presence of loss. It is almost non-achievable for current protocols in the practical systems. Assuming the infinite-size keys and ideal conditions (such as unit detector efficiencies, zero dark count rates, zero intrinsic error, unit error correction efficiency, and zero excess noise), the maximum rate of the CV-QKD protocol (the red solid line) scales as T/ln4, which is 1/2 of the PLOB bound [51].

If taking the finite-size effect and the non-ideal factors of physical devices into account, the secret key rate of the practical CV-QKD systems will be much lower. As shown in Figure 7, considering the non-ideal condition, the finite secret key rate of the CV-QKD system using RSEC can achieve $3.28\times 10^{-2}∼1.652\times 10^{-1}$ bits/pulse at 10∼27 km, which is about 0.115 of the PLOB bound. However, the system using SEC has a lower rate, which is just about 0.064 of the PLOB bound, at $7.1\times 10^{-3}∼9.22\times 10^{-2}$ bits/pulse.

The previous experimental results indicate that the proposed RSEC protocol is clearly advantageous. It significantly improves the quantization and reconciliation efficiency of SEC, which enables the CV-QKD system to achieve a higher secret key rate and a longer secure transmission distance. Overall, our work provides a better candidate for the application of the CV-QKD system.

## 5. Conclusions

In this research, we analyzed the strategy of the SEC protocol and proposed modifications to improve its anti-noise ability by performing a random orthogonal rotation on the correlated raw data and deducing a slice estimator. The experimental comparisons of the original SEC protocol and the proposed RSEC protocol show that the modifications can reduce the information loss of the quantization and release the performance limitation of SEC at the relatively low SNR.

Accordingly, both the secret key rate and the range of CV-QKD are increased. In order to accomplish the reconciliation of the raw data in CV-QKD, we implemented the RSEC protocol by combing with the polar codes. The reconciliation efficiency of RSEC protocol achieved above 95% when the input scale adopted 16 Mb. Both theoretical and experimental analysis showed that this is a more suitable reconciliation scheme for a practical CV-QKD system. 

## Figures and Tables

**Table 1:** Comparison of the reconciliation efficiencies between RSEC and some representative reconciliation works.

SNR	Reconciliation Efficiency β			
	Ref. [33] ${}^{a}$	Ref. [23] ${}^{b}$	Ref. [29] ${}^{c}$	This Work
3	94.1%	79%	88.7%	94.85%
5.12	94.4%	–	–	95.53%
7	–	84%	–	95.60%
14.57	95.8%	–	–	95.02%

${}^{a}$ The slice number and error correction codes adopted in [33] are not reported in detail; ${}^{b}$ It implements the four-slice and five-slice SEC with the LDPC for blocks of $2\times 10^{5}$; ${}^{c}$ It implements the four-slice SEC with the LDPC and Bose–Chaudhuri–Hocquenghem (BCH) for blocks of $2\times 10^{5}$ in a 25 km all-fiber CV-QKD system.

## Data Availability

Data is contained within the article.

## Appendix A

The mutual information between Alice and Bob $I_{AB}$ can be calculated by using Shannon’s channel capacity [29],

(A1) $$I_{AB}=\frac{1}{2}log_{2}\left(1+S_{nr}\right)=\frac{1}{2}log_{2}\left(\frac{V+\chi_{total}}{1+\chi_{total}}\right),$$

where $V=V_{A}+1$, $V_{A}$ represents Alice’s modulation variance, and $\chi_{total}=\chi_{line}+\chi_{hom}/T$ represents the total noise between Alice and Bob as previously defined.

The Holevo bound on information available to Eve is given by

(A2) $$\chi_{BE}=G\left(\frac{\lambda_{1}-1}{2}\right)\;+\;G\left(\frac{\lambda_{2}-1}{2}\right)\;-\;G\left(\frac{\lambda_{3}-1}{2}\right)\;-\;G\left(\frac{\lambda_{4}-1}{2}\right),$$

where $G\left(x\right)=\left(x+1\right)log_{2}\left(x+1\right)-xlog_{2}\left(x\right)$, and the symplectic eigenvalues $\lambda_{1,2,3,4}$ are given by

(A3) $$\lambda_{1,2}^{2}=\frac{1}{2}\left(A\pm \sqrt{A^{2}-4B}\right),\lambda_{3,4}^{2}=\frac{1}{2}\left(C\pm \sqrt{C^{2}-4D}\right),$$

with

(A4) $$A=V^{2}\left(1-2T\right)+2T+T^{2}{\left(V+\chi_{line}\right)}^{2},B=T^{2}{\left(V\chi_{line}+1\right)}^{2},$$

(A5) $$C=\frac{V\sqrt{B}+T\left(V+\chi_{line}\right)+A\chi_{hom}}{T(V+\chi_{total})},D=\frac{V\sqrt{B}+B\chi_{hom}}{T(V+\chi_{total})}.$$

When $N_{data}>10^{4}$, the finite-size offset factor $\Delta (N_{data})$ can be approximated as follows [18],

(A6) $$\Delta \left(N_{data}\right)\approx 7\sqrt{\frac{log_{2}\left(2/\epsilon \right)}{N_{data}}},$$

where ϵ is the security parameter.
